# Treatment of Insanity

**Published:** 1813-08

**Authors:** 


TREATMENT *of* INSANITY *by* MESSRS. TARDY *and* LUCETT, *communicated by* MR. TARDY, *Surgeon, of Marchmont-street, to* DR. FOTHERGILL.

SIR,

THE new method of curing insanity so laudably countenanced by His R. H. the Duke of Kent, and a committee of noblemen and gentlemen, having created considerable interest, I have been induced to transmit to your Journal a detailed report of the first case in which it was tried, conjointly by myself and Mr. Lucett, the gentleman who communicated to me this particular plan of treatment.

Various advertisements upon this subject appeared in the newspapers during 1811 respecting this case, inserted, however, without my knowledge, previously to which the following lowing report was sent, by Mr. Lucett, to the late Mr. Perceval, Chancellor of the Exchequer, signed by myself and by that gentleman.

*Case of Mr. Lucett's Process as performed upon Mr. Morgan, Editor of the Dublin Correspondent Newspaper.*

Monday, Sept. 23d, 1811.—I had been informed that Mr. M. had labored under a state of insanity for eighteen months, and had been attended by some eminent gentlemen in that practice. As far as my experience and observation of the case went, it had become one approaching to idiotism blended with melancholy: there was no mania ferox. This patient had very bad nights, getting up frequently to sing, and troubled with frequent inclination to go to stool. On the first application, the pulse was lowered in number, but rendered at the same time fuller by it. The patient was put to bed immediately after the operation, which was about ten o'clock at night, and had a bason of white wine whey. From the report of the attendant he slept profoundly, without awaking, from eleven o'clock that night until six o'clock next morning, when he was awoke by Mrs. M. going into his room, but went off to sleep again: he was awoke again about nine o'clock, and took his breakfast: he went to sleep again, and slept until two o'clock in the afternoon, when he was again awoke for his dinner. When I saw him at that time, he appeared refreshed, and was not so troublesome as when I saw him before, nor did I hear him attempt to sing, which he was continually doing the evening previous to this, and seemed more calm.

Sept. 24th.—Mrs. M.'s report to-day is, that he slept without interruption, was more tractable, and had not sung during this day.

25th.—Report the same as to sleep, &c. &c.

26th.—Report the same; sleep very good; diet, &c. as usual.

27th.—Report the sleep as before, but he had become somewhat more troublesome; would not keep up his stockings; and had begun to sing this evening.

28th.—Repeated the operation. He did not go to sleep so soon after the repetition of the process this night as at first, but he slept from ten o'clock until nine o'clock, when he was awoke for his breakfast. Mrs. M. reports that he was more rational through the day than previously, and that he did not commit any glaring act of inconsistency, nor did he sing, but went off to sleep again, and awoke for his dinner.

29th.—Report: slept soundly from nine o'clock this night until nine o'clock in the morning, when he was awoke for his his breakfast, and considered by Mrs. M. as much improved with regard to any errors or extravagance of mind. Mrs M. reports likewise that he was more particular in regard to his dress than she had observed him since the commencement of his malady, as he expressed a particular wish to have his coat brushed before he went out, and inquired for and wore his gloves, which he had not done before: he sung once this evening, but it was by particular desire of one who was not so well aware of his state of mind.

30th.—Report as to sleep the same; eat extremely hearty.

Oct. 1st to the 2d inclusive,—Slept composed from eleven o'clock (during a considerable noise in the neighbourhood) until nine o'clock on the next morning. Mrs. M. has not noticed any irrational word from him during these days: he dressed himself entirely, putting on his cravat himself without help, which he had not done previous to this time, and likewise his boots, which he had not worn for some time before. Mrs. M. reports that she had been obligated to dress him before when going out with him, but he appeared dressed correctly when she wished to take him out with her. He was extremely anxious to walk out with Mrs. Morgan on these days.

E. Tardy.

To which Mr. Lucett subjoined his signature.

The gentleman whose case is described in the above report was about 44 years of age, inclining to corpulency; had been of a cheerful disposition, and his avocations being of a public nature, he was led much into company, when he sometimes unavoidably exceeded the bounds of temperance.* He was subject to hæmorrhoidal affections, and for about nine or ten years previous to the first accession of his malady, he had had repeated attacks of the gout, which totally disappeared after the accession of insanity. For some time before the first appearance of his mental derangement, he complained of a constant pain in the back of his head, which was supposed to arise at that time from a translation of gout. On the superior part of the right parietal bone he had the cicatrix of an injury he received early in life, but I could never learn that it had been attended with any bad symp-

* Through life he had been an affectionate husband and father. Some months previous to the accession of his malady he lost a favorite child: it was conceived of consequence to open the child's head after death: he insisted upon being present, and Mrs. M. was of opinion that his symptoms of derangement advanced more rapidly from that time. About this time, likewise, he experienced a disappointment in his hopes of remuneration for his professional services, and his circumstances in consequence became considerably embarrassed.

toms, toms. The approaches to insanity were gradual, various slight symptoms of an erroneous judgment showing themselves for some time previously.

These, as nearly as I could learn, were the principal facts that tended to elucidate the causes and nature of this gentleman's malady.

It will be observed by the report, that his state of rest was restored, and his disposition to sing and tear books, &c. during the day, was removed. His erroneous train of thoughts still remained after the process, though somewhat diminished; such as his constant regret at his reduced circumstances, at the smallness of his house, and his desire to have a larger one, with a number of servants to attend him, although his style of living was as good as his means could justify.

The treatment employed by Mr. Lucett and myself upon this gentleman, were warm immersion and warm affusions on the head, conducted in such a manner, by a considerable column of water, as to produce a slight concussion upon the shaven vertex. The bath was at first 93°, and subsequently increased to 107 and 8. He was kept in the bath about four minutes in the first trial, and afterwards from thirty to forty minutes.

The treatment was continued a short time after the above report had been sent to Mr. Perceval; but owing to an incautious and sudden increase of the temperature of the bath, a considerable and dangerous discharge of blood took place from the hæmorrhoidal vessels, to the amount of ten ounces per day. At first I was inclined to hope that this discharge might prove critical and beneficial, more especially as his hæmorrhoidal affection ceased suddenly previous to his first attack: but those hopes were disappointed; for after three days continuance of this profuse discharge, he evidently became worse, symptoms of fatuity and loss of memory being clearly perceptible. Under those circumstances, I objected to the further continuance of the bath, &c. This discharge was the more to be regretted, as unequivocal symptoms of amendment had shown themselves from the moderate application of the above means.

There were no other means used in this case but the above; indeed he had been attended by many eminent gentlemen in that practice, and I considered medicine as superfluous, and as likely to be injurious in his case.

In an establishment in which I am about to engage for the reception of insane persons, I propose to try this process with some variations on this gentleman again; and the results upon him, as well as upon some others, I will transmit to you for publication.

It may be expected that something should be said of the *modus operandi* of this application; but I apprehend the limited number of cases that have hitherto been tried, are not sufficient to warrant any just conclusions. The bath, warm and cold, has been so long used in cases of insanity, that its importance need not be dwelt upon. From the successful result of its application in two or three recent cases, it may be concluded (supposing those patients to have had the bath in the common mode) that the benefit arises from the concussion produced; and it may be effected by cold affusion likewise, while the patient is kept in a warm bath, the affusion being conducted in such a manner as to prevent the cold water from running over the patient's body, which mode of applying it I have recently tried with considerable benefit in a case of mild continued insanity; but from the result of two of the cases above alluded to, and the testimony of many writers on this subject, as well as some cases I have lately seen, it appears that a bath of very high temperature almost always allays the violent paroxysms of mania. The warm bath entirely overcomes the preternatural muscular force which they exert, and by the aid of affusion, if continued a sufficient length of time, produces syncope, and afterwards a propensity to sleep; and yet under these circumstances there is a considerable determination to the surface of the head and face, both becoming highly efflorescent. These appearances would lead one to conclude that some increased action was induced in the head, thereby overcoming any unequal distribution or impeded circulation of the blood in it. It may be supposed from those effects and appearances, that the affusion conducted in this manner must be extremely hazardous in those cases which arise from external injury of the head: in such patients the warm bath with cold affusion, and the addition of ice, as I have lately experienced, promises more favorable results; and I suspect this latter mode to be the means so much celebrated in a recent American practice. In those cases in which warm immersion and affusion were used conjointly, profuse diaphoresis was induced, which in one instance appeared to be attended with considerable benefit; in others it had a contrary effect.

These are the principal effects which I have observed from this mode of applying the bath in this malady. How far, and in what species of insanity it will prove of the greatest benefit, remains to be ascertained hereafter; as likewise how far it will secure the wretched objects of these attacks from relapses.

*Marchmont-street, Russell-square,* E. TARDY.

*July* 20, 1813.

COLLEC-

